# Replacing Soybean Meal with Hemp Leaves with Very Low THC Content in the Diet for Dairy Cows: Impact on Digestibility, Nitrogen Use Efficiency and Energy Metabolism

**DOI:** 10.3390/ani15111662

**Published:** 2025-06-04

**Authors:** Jessica Schwerdtfeger, Solvig Görs, Björn Kuhla

**Affiliations:** Research Institute for Farm Animal Biology (FBN), Wilhelm-Stahl-Allee 2, 18196 Dummerstorf, Germanygoers@fbn-dummerstorf.de (S.G.)

**Keywords:** respiration chamber, methane, Santhica, urine, rumen fluid

## Abstract

The aim was to investigate the suitability of Santhica 27 industrial hemp leaves as a protein source in dairy cow nutrition. Twelve Holstein dairy cows received a total mixed ration (TMR) containing 7.4% industrial hemp leaves (HEMP) and a TMR containing 3.5% soya extraction meal (CON) in a crossover design. Cows were kept in a free-stall barn for 2 weeks to measure feed intake, milk yield and sample plasma, ruminal fluid, and urine. In week 3, cows were housed in a respiration chamber to measure gas exchange, urine, and feces excretions. In the first two weeks, cows of the HEMP group rested longer but spent less time ruminating. Feeding the HEMP diet reduced dry matter intake (DMI), milk yield, and urinary N-metabolite concentrations and tended to lower total N-excretion, milk fat, and lactose concentrations. During the stay in the respiration chamber, DMI, milk yield, apparent digestibility, and crude protein degradability were similar between groups, but feeding the HEMP diet tended to reduce methane yield. In conclusion, Santhica 27 hemp leaves are a suitable protein source for dairy cows as they have no negative effects on animal health, apparent digestibility, and crude protein degradability. Nevertheless, inadequate adaptation to the diet reduces feed intake and milk yield.

## Simple Summary

We studied the suitability of industrial hemp leaves with very low tetrahydrocannabinol content as an alternative protein source for dairy cow nutrition, relative to soybean meal, on performance traits, apparent digestibility, and animal health and behavior. Our findings show that dried Santhica 27 hemp leaves can be a suitable protein source for dairy cows, as animal health and apparent digestibility of the diet are not negatively affected; however, inadequate adaptation to the diet can result in a decreased feed intake and milk yield.

## 1. Introduction

Imports of protein-rich animal feed such as soybeans and soy meal from North and South America to Europe amounted to approx. 33 million tons in 2022, with German imports accounting for 3.3 million tons of soybeans in the same year [1]. Germany’s carbon footprint amounts to 0.89 t CO_2_ equivalents/t of soy imported from Brazil, whereas the European Union’s average is only 0.77 t CO_2_ equivalents/t [2]. The greatest contributions to these footprints result from land use changes and domestic and marine transports [2]. In order to reduce the CO_2_ and environmental footprint from soy imports, more domestically grown protein crops should be used in the feeding of livestock in future. Legumes may be alternative protein feedstuffs, but their cultivation requires three- to five-year cultivation breaks. Industrial hemp (*Cannabis sativa*) is a non-legume plant, and varieties listed in the Common Catalogue of Varieties of Agricultural Plant Species that contain less than 0.3% Δ -9 tetrahydrocannabinol (THC) can be legally cultivated in the European Union. Hemp seed by-products, e.g., hempseed cake or hemp meal, can perfectly replace soybean meal in rations for heifers and dairy cows without negative effects on performance or meat quality [3,4,5], and may offer an 8 to 11% CO_2_ footprint reduction potential [6]. However, hempseed press cake is also a protein source for human consumption [7].

To avoid this food–feed competition, leaves and flowers or their residue from cannabinoid extraction—spent hemp biomass (SHB)—could be a decent feed for ruminants. Chemical analysis revealed that SHB has a nutritive quality and crude protein (CP) content similar to alfalfa [8] which can be referred to the crude concentration of flowers and leaves ranging between 13% and 24% of dry matter (DM) [9]. A feeding level of 13% SHB pellets to dairy cows decreased intake and lying time, whilst milk yield, milk constitutes, DM, and CP digestibility, as well as methane emissions, were not affected compared to counterparts fed a comparable amount of alfalfa pellets [8]. However, feeding SHB pellets resulted in a lower urine volume and urinary N excretion but comparable urinary urea, purine derivatives, and creatinine concentration, suggesting a higher nitrogen (NUE) and feed energy utilization efficiency (EUE) in SHB-fed cows [8]. Yet, the plasma protein, urea, glucose and non-esterified fatty acids (NEFA) concentrations, the ruminal ammonia concentrations, or the molar portion of short-chain fatty acids (SCFA) were not affected by feeding SHB to dairy cows [8,10], calling for further studies to elucidate the mechanism of improved NUE and EUE with SHB feed.

If industrial hemp leaves are not extracted and depleted of bioactive cannabinoids, they may contain psychoactive THC [9]. The incubation of hemp leaves and stalks with ruminal fluid in vitro reduced methane production without negative effects on feed degradability and the volatile fatty acid pattern, but the methane mitigating effect was not attributed to THC but to flavonoids [11]. Feeding 35 g whole-plant industrial hemp per day (d) to Holstein bull calves (average dose of Δ9 tetrahydrocannabinolic acid (THCA-A) 117.4 mg/d) increased the plasma THCA-A concentration from 1 to 10 ng/mL after 24 h of ingestion, indicating the bioavailability and absorption of THC compounds by ruminants [12]. In addition, the inclusion of 25 g industrial hemp prolonged the lying time by 42 min per d in castrated male Holstein cattle [13]. Furthermore, THC can exert a negative impact on cow health. Wagner et al. (2022) reported that feeding 1.68 kg DM of hemp silage containing 1254.7 mg/kg of DM THC to dairy cows resulted in bradypnea and bradycardia in some animals and that THC is transferred into their milk, exceeding the acute reference dose for humans [14]. These unwanted effects exclude its use as feed for dairy cows. However, the hemp variety Santhica 27, listed in the Common Catalogue of Varieties of Agricultural Plant Species of the European Union, contains particularly low THC and cannabidiol (CBD) concentrations [15], suggesting that feeding leaves from this variety exerts less unwanted side-effects and could improve NUE and EUE, like the feeding of SHB does. Therefore, the aim of this study was to investigate the impact of feeding dried Santhica 27 leaves compared to soybean meal on feed intake and feeding behavior, milk yield and constitutes, digestibility, post-absorptive metabolite profile, and nitrogen and energy metabolism of dairy cows. We hypothesized that feeding dried Santhica 27 hemp leaves would not negatively affect health, dry matter intake (DMI) and milk performance, but reduce urinary nitrogen excretion and methane production and thus NUE and EUE of dairy cows.

## 2. Materials and Methods

The experimental protocol was evaluated by the ethical committee and approved by the Federal Office of Agriculture, Food Security and Fishery, Mecklenburg-Western Pomerania, Rostock, Germany (LALLF, permission no. 7221.3-1-028/22) and conducted in accordance with the ARRIVE guidelines (https://arriveguidelines.org/, download on 12 December 2024), the European Directive 2010/63/EU, and the German Animal Welfare Act. Personnel involved in sample preparation and analyses were blinded, except for the analyses of ruminal pH and ammonia concentrations. Personnel who determined the body constitution, collected blood, urine and rumen fluid samples, prepared the feed, fed the cows, or were involved in respiration chamber experiments were not blinded.

For this study, 12 German Holstein dairy cows in the first lactation were selected from the herd of the Experimental Facility for Cattle (FBN, Dummerstorf, Germany) and entered the experimental trial in 3 blocks of 4 animals each. At the beginning of the experimental trial, the cows were 227 ± 29 days in milk and 116 ± 45 days in gestation (mean ± standard deviation (SD)). Four cows in each block were randomly assigned to two isoenergetic and isonitrogenous formulated diets in a 2 × 2 crossover design. The hemp group (HEMP, *n* = 12) received a total mixed ration (TMR) containing 7.4% (on DM basis) dried industrial hemp leaves of the low THC variety “Santhica 27”. The control group (CON, *n* = 12) were fed a TMR containing 3.5% soya extract meal (on the DM basis). Each experimental feeding period lasted for 21 d, followed by a washout period of 14 d (Supplementary Figure S1). In the first two weeks of each feeding period, cows were housed in a free-ranging barn of the experimental facilities at FBN and had ad libitum access to feed and water. Cows were fed at 0730 h and 1430 h. The daily feed and water intake of each individual cow was measured by the Roughage Intake Control system (RIC, Insentec B. V., Marknesse, The Netherlands). Feed samples were taken twice a week, and the nutrient composition was analyzed by the Landwirtschaftliche Untersuchungs- und Forschungsanstalt (LUFA GmbH, Rostock, Germany) (Table 1). In addition, feed samples were dried at 60 °C for 24 h, ground and then subjected to further drying at 103 °C for 4 h to determine the DM content once a week. The gross energy of the feed was determined by the combustion of approximately 0.5 g of a dried and ground (1 mm particle size) feed sample in a bomb calorimeter (IKA C200, IKA-Werke GmbH & Co. KG, Staufen, Germany), using a cotton thread (C 710.4, IKA-Werke GmbH & Co. KG) and a combustion bag (C 12 A, IKA-Werke GmbH & Co. KG) as combustion aids. Cows were milked twice daily at 0630 h and 1700 h, and the milk yield was recorded. Due to technical problems, the milk yield and the feed intake of 5 animals of the HEMP group and 2 animals of the CON group on day 0 had to be excluded from the calculation of the weekly mean. Milk samples were taken twice a week by pooling a sample of the evening milking with a sample of the subsequent morning milking, according to the respective milk yield. The body weight (BW) of the cows was determined twice daily after milking using a walk-through scale and a weekly mean of the BW and the metabolic BW (mBW = BW^0.75^) was calculated. In addition, the BCS was determined weekly according to Edmonson et al. (1989) [16]. The back fat thickness (BFT) was determined once a week using a Titan Ultrasound System with a linear probe (L52/10-5 MHz Transducer, Sonosite Inc., Bothell, Washington, DC, USA), as described by Schröder and Staufenbiel (2006) [17].

To assess the health of the cows, the respiratory rate was recorded daily prior to the morning feeding by counting the flank movements. In addition, the rectal temperature was measured daily prior to the morning feeding. To investigate the influence of diet on animal behavior, cows were equipped with a Moomonitor+ collar (Dairymaster, Tralee, Ireland) containing a 3D accelerometer to record feeding, rumination, and resting time. One animal in the CON group had to be excluded from the evaluation of the feeding, resting and rumination times due to technical issues with the equipment.

On d 0, 7, and 14 of each feeding period, blood samples were collected from the jugular vein at 0700 h in 9 mL EDTA-containing tubes (Sarstedt AG & Co. KG, Nümbrecht, Germany) and immediately placed on ice. Subsequently, blood samples were centrifuged at 1.570× *g* for 20 min at 4 °C and the obtained plasma was stored at −80 °C for further analysis.

Rumen fluid was collected on d 14 of each period, 2 h after the morning feeding using an oral stomach tubing [18]. Subsequently, rumen fluid was immediately placed on ice and filtered through a sieve with a pore size of 1 mm. In addition, on d 14 of each feeding period, spot urine samples were collected 2 h after the morning feeding by stimulating the ventral vulva region. Urine samples were filtered using filter paper to remove coarse particles and stored at −80 °C for further analysis.

On d 14, after rumen fluid and urine samplings, cows were transferred to the Animal Facility Center at FBN to measure gas exchange and the apparent digestibility for 4 d (d 14–18) at 15 °C in respiration chambers [19]. Cows were habituated to the respiration chambers prior to the start of the experiment for a minimum of 3 d until all cows laid down, ruminated and consumed feed and water as described by Müller et al. (2021) [20]. The BW of the animals was determined directly before entering and after exiting the chamber to calculate a mean mBW. To monitor the health of the animals, the heart rate was determined daily by auscultation with a stethoscope, in addition to rectal body temperature and respiratory rate measurements. The animals had ad libitum access to water and the daily water intake was recorded using water meters. In the chamber, cows were fed ad libitum at 0730 h and 1645 h and milked daily at 0630 h and 1700 h. The daily milk yield was recorded and on d 15, a milk sample was taken, as described above, for the analysis of milk constituents. The amount of ingested feed was determined by back weighing the feed residues before the morning feeding.

### 2.1. Analysis of Milk Samples

Milk fat, protein, lactose and urea concentrations were analyzed by infrared spectroscopy at the State Inspection Association for Performance and Quality Testing, Mecklenburg-Western Pomerania e.V. (LKV, Güstrow, Germany). Milk composition was used to calculate the fat–protein ratio and energy-corrected milk yield (ECM) using the following equation:

ECM=kg milk yield∗ ((0.38∗ % Fat+0.21∗ % Protein+1.05)/3.28)

One animal in the HEMP group had to be excluded from the evaluation of the milk constitutes in experimental week 3 due to mastitis.

### 2.2. Rumen Fluid Analysis

Directly after the collection of the rumen fluid, the pH value was measured using a pH meter (HI 208, Hanna Instruments Deutschland GmbH, Vöhringen, Germany). Subsequently, the ammonia concentration was determined using the modified Conway microdiffusion method in fresh rumen fluid in triplicate, as previously described by Prahl et al. (2023) [21]. For the analysis of SCFA, rumen fluid was centrifuged for 10 min at 4000 rpm at 4 °C, and 2.5 mL of the supernatant was mixed with 1 mL of iso-caproic (43 mM) acid, dissolved in 17% phosphoric acid, and centrifuged again for 10 min at 13,000 rpm at 4 °C. The obtained supernatant was frozen at −20 °C until further analysis. The concentration of SCFA was determined in the supernatant via gas chromatography (GC-17A, Shimadzu Corporation, Kyoto, Japan) in quintuplicate using a capillary column (ROTI^®^Cap-FFAP, 25 m × 0.25 mm, Carl Roth GmbH + Co. KG, Karlsruhe, Germany) maintained at 90 °C and a flow of 0.64 mL/min. The flame ionization detector was maintained at 220 °C.

### 2.3. Analysis of Plasma Metabolites

Plasma metabolites were analyzed at a clinical chemistry analyzer (ABX Pentra 400, HORIBA Medical, Kyoto, Japan) using the following kits: NEFA: NEFA-HR (2), FUJIFILM Wako Chemicals Europe GmbH, Neuss, Germany, glucose: ABX Pentra Glucose HK CP, urea: ABX Pentra Urea CP, albumin: ABX Pentra Albumin CP, uric acid ABX Pentra Uric Acid CP, and creatinine: ABX Pentra Enzymatic Creatinine CP (all HORIBA ABX, Montpellier, France). The plasma NEFA concentrations determined on day 0 of two CON cows and one HEMP cow were detected as outliers and thus excluded from further analysis.

### 2.4. Urine Sample Analysis

The total nitrogen (N) content of urine samples was determined in triplicate using an element analyzer (FlashEA 1112 NC Analyzer, Thermo Quest, Milan, Italy). Non-urea N metabolites were measured using high-performance liquid chromatography (HPLC) (1200/1260 infinity Series; Agilent Technologies, Waldbronn, Germany). Frozen urine samples were thawed on ice, homogenized, and centrifuged at 13.000 rpm at 4 °C for 5 min. The supernatant was ten-fold diluted with phosphate buffer (20 mM, pH 6.5) and kept at 4 °C until analysis. A total of 5 µL of the diluted urine sample were separated on a 250 × 4.6 mm Synergi 4 µm Hydro-RP 80 Å column protected by a corresponding 4 × 3 mm pre-column (both Phenomenex Inc., Aschaffenburg, Germany) at constant 25 °C. The elution was performed, according to Müller et al. (2021) [20], with phosphate buffer (20 mM, pH 6.5) modified by a gradient with acetonitrile ranging from 0 to 10% and from 5 to 25 min, at a flow rate of 1 mL/min. Metabolites were detected by a UV detector and quantified at 230 nm, with the exception of allantoin and creatine, which were quantified at 210 nm. For the measurement of urinary urea concentration, the supernatant was diluted 50-fold with ultrapure water. A total of 20 µL of the diluted urine sample were separated on a 300 × 7.8 mm Rezex RCM-Monosaccharide column protected by a 4 × 3 mm Carbo-Ca pre-column (both Phenomenex Inc., Aschaffenburg, Germany) at constant 60 °C and detected by refractive index according to Müller et al. (2021) [20]. Ultrapure water at a 0.5 mL/min flow rate was used as an eluent. All metabolites were quantified against multipoint calibrated external standards and analyzed in single measurements.

### 2.5. Energy and Nitrogen Use Efficiency

The EUE was calculated by dividing milk energy output by energy intake. Milk energy output (MJ/d) was estimated as described by Erdmann et al. (2019) [22]:

$$\begin{array}{cc} Milk\;energy & output\left(MJ\right)\\ & \;=(0.038*milk\;fat\left(\frac{g}{kg}\right)+0.024*milk\;protein\left(\frac{g}{kg}\right)\\ & \;+0.017*lactose\left(\frac{g}{kg}\right))*milk\;yield\;(kg) \end{array}$$

Metabolizable energy intake (MEI) was calculated as follows:

$$MEI\;\left(MJ\right)=ME\;\left(\frac{MJ}{kg\;of\;DM}\right)*\;DMI\;(kg)$$

The total N content of the feed was determined using the Kjeldahl method and the N intake with feed was calculated by multiplying DMI with N content of the feed. The total N content of the feces samples (see below) was determined in duplicate using an element analyzer. In addition, the N content of milk was calculated by dividing the milk protein content by 6.38. Then, the N excretion of feces, urine and milk was calculated.

The NUE was calculated as follows:

NUE=Milk N excretion (g)/N intake (g)

For the calculation of the urinary N excretion, the N content of the urine collected on d 14, and the urine volume measured on d 15–18 was used.

### 2.6. Excreta Collection and Apparent Digestibility

To assess the apparent digestibility, the average daily feed intake of a 4 d period (d 15 to 18) was calculated. To quantify the daily amount of urine and to separate the urine from the feces, a urinal, connected with a hose to a canister, was glued to the cows. In 2 animals (1 CON, 1 HEMP), the urinal detached during the measurement period and the amount of excreted urine could not be determined. Feces were collected manually multiple times throughout the day during the stay in the respiration chamber and stored at 4 °C in order to reduce volatile N losses. Feces collected within 24 h were pooled and weighed, and a sample was taken and stored at −20 °C until further analysis. To determine the DM content, fecal samples were dried at 60 °C for 24 h, ground (0.7 mm particle size) and further dried at 103 °C for 4 h. In addition, feed and feces subsamples were incinerated at 550 °C for 2 h to determine the crude ash and the organic DM content. The gross energy of the feces was determined by the combustion of approximately 0.5 g of dried and ground (0.7 mm particle size) samples in a bomb calorimeter (IKA C200, IKA-Werke GmbH & Co. KG, Staufen, Germany) as described for the feed samples. Gross energy was determined in single measurements. The apparent digestibility of the organic matter (OM) was calculated by subtracting the excretion of OM from OM intake divided by the OM intake. In addition, the CP degradability was computed by N intake with feed minus the N excretion with the feces divided by the N intake with feed. Furthermore, the apparent energy digestibility was calculated by subtracting the energy excreted with the feces from the energy ingested with the feed divided by the energy intake.

### 2.7. Indirect Calorimetry and Methane Production

Gas exchange and methane production was measured over a 48 h period, starting on d 16 at 0800 h and ending on d 18 at 0800 h. The airflow through the respiration chamber was set to 30 m^3^/h and was measured by a differential pressure-type V-Cone flow meter (McCrometer, Hemet, CA, USA). The concentrations of CO_2_ and CH_4_ were measured by infrared absorption (SIDOR, Sick AG, Waldkirch, Germany) and the concentration of O_2_ was analyzed paramagnetically (SIDOR, Sick AG, Waldkirch, Germany) at 6 min intervals. Mean gas recovery rate from the four respiration chambers was 99.7%. Total CO_2_ production is composed of CO_2_ from fermentative (CO_2 ferm_) and metabolic (mCO_2_) processes. According to Chwalibog et al. (1996) [23], CO_2 ferm_ was calculated using the following equation:

$${{CO}_{2}\;}_{ferm}\;\left(L\right)=1.7\;*\;{CH}_{4}\;(L)$$

The mCO_2_ was computed by subtracting CO_2 ferm_ from total CO_2_. The metabolic respiratory quotient (mRQ) was calculated by dividing mCO_2_ by O_2_. The net fat oxidation (FOX) and carbohydrate oxidation (COX) was calculated according to Frayn (1983) [24], using the following equations:

$$FOX\;\left(g\right)=1.67*\;O_{2}-1.67*\;{mCO}_{2}-1.92*\;N_{u}\;COX\;\left(g\right)=4.55*\;m{CO}_{2}-3.21*\;O_{2}-2.87*\;N_{u}$$

Furthermore, the heat production (HP) was calculated according to Brouwer (1965) [25] using the following equation:

$$HP\left(kJ\right)=16.18*O_{2}+5.02*{CO}_{2}-2.17*{CH}_{4}-5.99*N_{u}$$

Urinary N excretion (N_u_) was calculated from the N content of the urine measured on day 14 and the mean urine volume measured on days 15–18.

The daily mean of the mRQ and CH_4_ recordings was calculated. In addition, a 24 h mean of HP, FOX, and COX were computed. Mean CH_4_, HP, FOX, and COX were normalized to DMI.

Energy balance (EB) was calculated according to Erdmann et al. (2019) [22]:

$$EB\;(MJ)=MEI\;(MJ)-HP\;(MJ)-[ECM\;\left(kg\right)*\;0.00314\;MJ/kg].$$

One animal was excluded from the analyses of the 4 day measurement period in the respiration chamber in both feeding periods, as this cow consumed less than 0.1 kg/mBW. One animal in the HEMP group had to leave the chamber before the end of the 4 day measurement period because it did not lie down and thus was excluded from the statistical analysis.

### 2.8. Statistical Data Analysis

The sample size required to prove the equivalence of the crude protein digestibility of the two rations was calculated using ‘sampleN. TOST()’ from the “PowerTOST” package (version 1.5.4) [26] in R Statistical Software (R core Team, 2021, version 4.4.2, assuming a coefficient of variation of 15%, a ratio of the means of 0.9, a lower and upper equivalence limit of 0.75 and 1.33, respectively, and a probability for the type I error of α = 0.05 and for the type II error of β = 0.2). In addition, the sample size for the methane reduction test was calculated using the ‘pwr.t.test()’ function from the R package ‘pwr’ (version 1.3.0) [27], assuming Cohen’s d = 1.0, and a type I error probability of α = 0.05 and a power = 0.8.

Statistical analyses were performed using R Statistical Software (v4.4.2; R Core Team, 2023 [28]). Datasets were checked for the presence of outliers using robust z scores (performance package, v0.11.0; Lüdecke et al. (2021) [29]) and visual inspection of boxplots. Data were analyzed with a linear mixed model (LMM, lmer function, lmerTest package, v1.1-29; Kuznetsova et al. (2017) [30]). Based on the experimental design, group (level: HEMP and CON), period (level: period 1 and period 2), sequence (level: hemp-soya or soya-hemp), and the block (levels: Block 1, Block 2 and Block 3) were defined as fixed effects and the animal ID was defined as random effect. For datasets containing multiple measurements within a feeding period, the time point (day/week) and the interaction with the group (group × time point) were also included as fixed effects. The residuals of the models were evaluated for normal distribution and homoscedasticity (check_normality and check_heteroscedasticity function, performance package, v0.11.0; Lüdecke et al. (2021) [29]). If the assumption of normality was violated, data were log2 transformed and re-evaluated. If normal distribution could not be achieved by transformation, a generalized mixed model was used and a gamma distribution was assumed (glmer function, glmmTMB package, v1.1.8; Brooks et al. (2017) [31]). Models without a constant error variance were used without further transformation as heteroscedasticity has only a marginal impact on model estimates [32]. Models were checked for singularity using the check_singularity function of the performance package (v0.11.0; Lüdecke et al. (2021) [29]). If singularity was detected, data were analyzed using a linear model including group (level: HEMP and CON), period (level: period 1 and period 2), sequence (level: hemp-soya or soya-hemp) and the block (levels: Block 1, Block 2 and Block 3) as fixed effects. If multiple measurements were analyzed, models also included the time point (day/week) and the interaction with the group (group x time point) were included as fixed effects. Pairwise differences were tested using the Tukey–Kramer test. For the fixed effect of interest, estimated marginal means and their standard errors (SE) were estimated. Effects and differences were considered significant at *p* < 0.05 and considered as trend at *p* < 0.10.

## 3. Results

### 3.1. Animal Behavior, Feed and Water Intake

**Figure 1:**
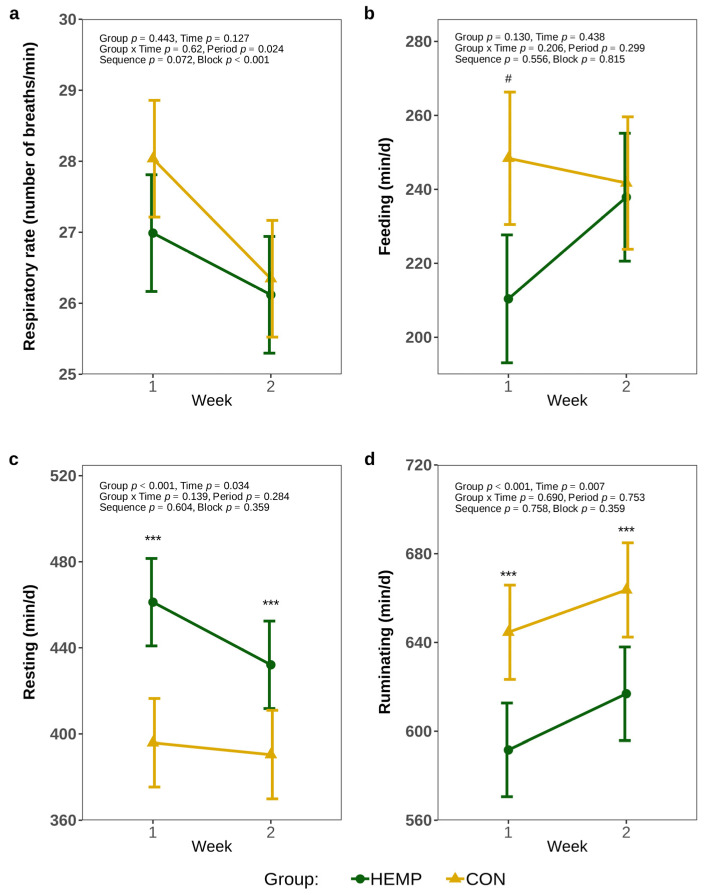
Respiratory rate (**a**), feeding (**b**), resting (**c**), and rumination (**d**) times per day of cows fed a diet containing 7.4% hemp leaves (HEMP, *n* = 12) or 3.5% soya extraction meal (CON, *n* = 12) in the first 2 weeks of the feeding period. # *p* < 0.1, *** *p* < 0.001.

The respiratory rate was comparable between groups throughout the feeding period (*p* > 0.1, Figure 1a). In the first week of experimental feeding, HEMP cows tended to spend 38 min less time per d feeding than CON cows (*p* = 0.053, Figure 1b). However, in week 2, no significant difference in the duration of feeding was observed between the groups (*p* = 0.841). The cows of the HEMP group rested 65 min/d longer in the first week and 42 min/d longer in the second week (Figure 1c). HEMP cows also ruminated 53 min/d less in the first week and 47 min/d less in the second week relative to CON cows (*p* < 0.001, Figure 1d).

**Figure 2:**
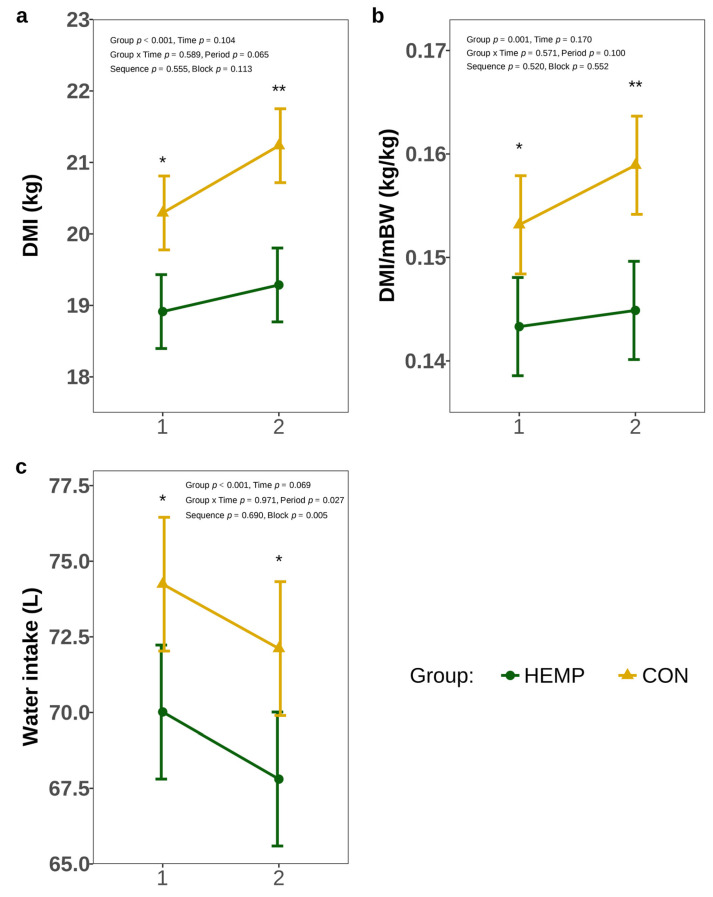
Dry matter intake (DMI) (**a**), dry matter intake per metabolic body weight (DMI/mBW) (**b**), and water intake (**c**) of cows fed a diet containing 7.4% hemp leaves (HEMP, *n* = 12) or 3.5% soya extraction meal (CON, *n* = 12) in the first 2 weeks of the feeding period. * *p* < 0.05, ** *p* < 0.01.

The DMI differed significantly between the groups (Figure 2a). In week 1, the HEMP group ingested 1.5 kg (*p* = 0.025) and in week 2, 1.9 kg less DM (*p* = 0.004) than the CON group. In addition, DMI/mBW of HEMP was significantly reduced compared to the CON group in weeks 1 and 2 (*p* < 0.05), Figure 2b). Furthermore, cows of the HEMP group drank 4.2 L less water in week 1 (*p* = 0.014) and 5 L less water in week 2 (*p* = 0.013, Figure 2c).

### 3.2. Animal Performance, Energy and N Use Efficiency

**Figure 3:**
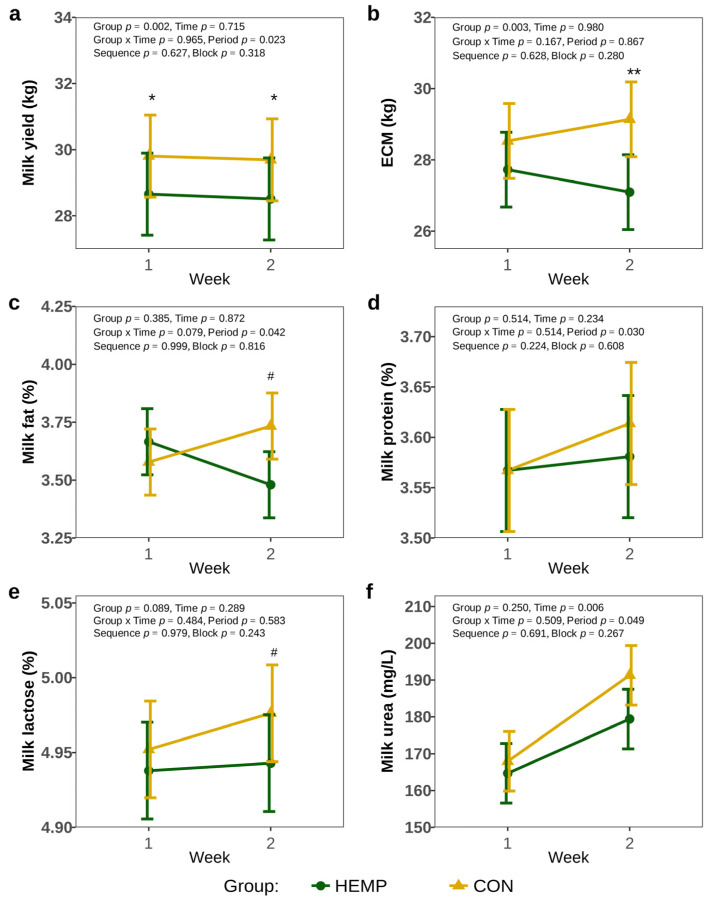
Milk yield (**a**), energy corrected milk yield (ECM) (**b**), milk fat concentration (**c**), milk protein concentration (**d**), milk lactose concentration (**e**), milk urea concentration (**f**) of cows fed a diet containing 7.4% hemp leaves (HEMP, *n* = 12) or 3.5% soya extraction meal (CON, *n* = 12) in the first 2 weeks of the feeding period. # *p* < 0.1, * *p* < 0.05, ** *p* < 0.01.

One week before the start of the experiment, milk yield did not differ between the groups (HEMP: 30.2 ± 1.68 kg, CON: 30.8 ± 1.68 kg, *p* > 0.8). Feeding cows a diet containing Santhica 27 hemp leaves reduced milk yield by 1.1 L/d in week 1, and 1.2 L/d in week 2, compared to a diet containing soya extraction meal (*p* < 0.05, Figure 3a). However, there was no difference in ECM between the two groups in week 1, but in week 2, ECM was 2 L/d lower in the HEMP compared to CON group (*p* = 0.002, Figure 3b). The analysis of milk protein and milk urea concentrations revealed no differences between groups (*p* > 0.1, Figure 3d,f). Furthermore, milk fat and milk lactose concentrations were comparable between the groups in week 1 (*p* > 0.1), but tended to be lower in HEMP group in week 2 (*p* < 0.1, Figure 3c,e).

**Figure 4:**
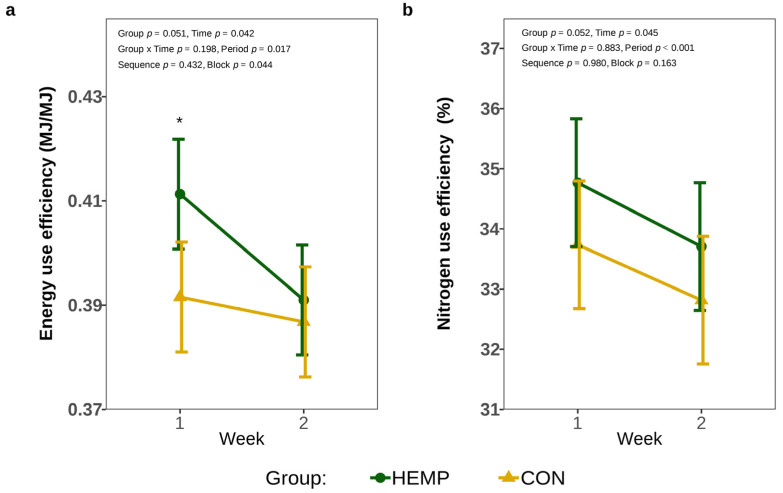
Energy utilization efficiency (EUE) (**a**) and nitrogen use efficiency (NUE) (**b**) of cows fed a diet containing 7.4% hemp leaves (HEMP, *n* = 12) or 3.5% soya extraction meal (CON, *n* = 12) in the first 2 weeks of the feeding period. * *p* < 0.05.

The inclusion of Santhica 27 leaves in the diet increased EUE by 4.8% in week 1 (*p* = 0.024); however, EUE was no longer affected by the diet after two weeks of hemp feeding (*p* = 0.618, Figure 4a). In addition, feeding the HEMP diet tended to affect NUE by approx. 1% (*p* = 0.052), but NUE decreased over time in both the CON and HEMP groups (*p* = 0.045; Figure 4b).

**Figure 5:**
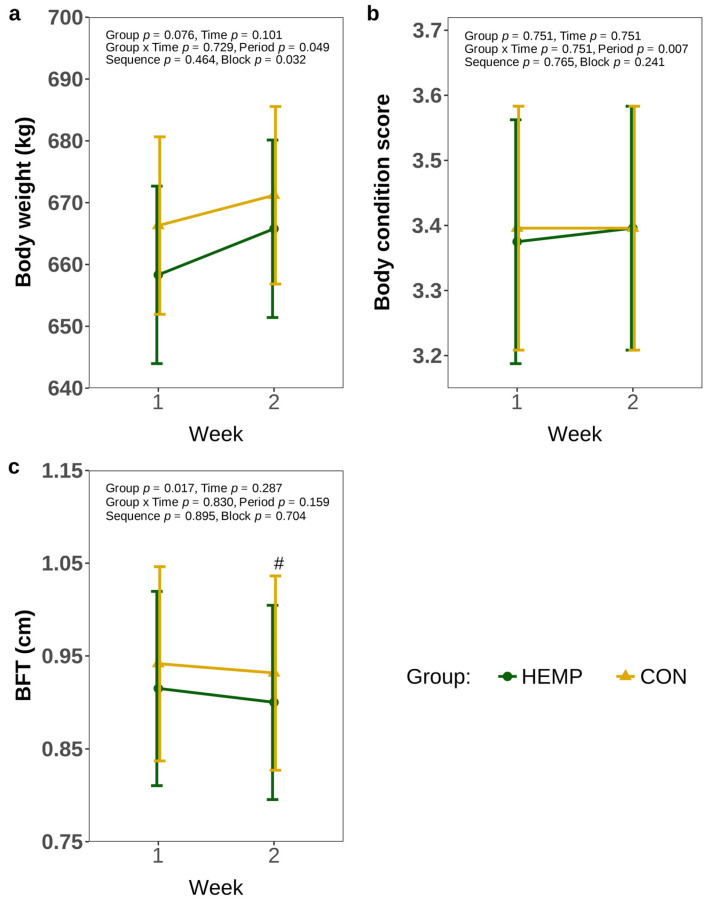
Body weight (**a**), body condition score (BCS (**b**), and back fat thickness (BFT) (**c**) of cows fed a diet containing 7.4% hemp leaves (HEMP, *n* = 12) or 3.5% soya extraction meal (CON, *n* = 12) in the first 2 weeks of the feeding period. # *p* < 0.1.

In addition, the BW did not change over time (*p* > 0.1) and was comparable between groups (*p* > 0.1, Figure 5a). Comparably, BCS did not differ between the groups or weeks of feeding (*p* > 0.1, Figure 5b). However, HEMP cows tended to develop a lower BFT compared to CON cows in week 2 (*p* = 0.061), while BFT was comparable between feeding groups in week 1 Figure 5c).

### 3.3. Plasma Metabolites

**Figure 6:**
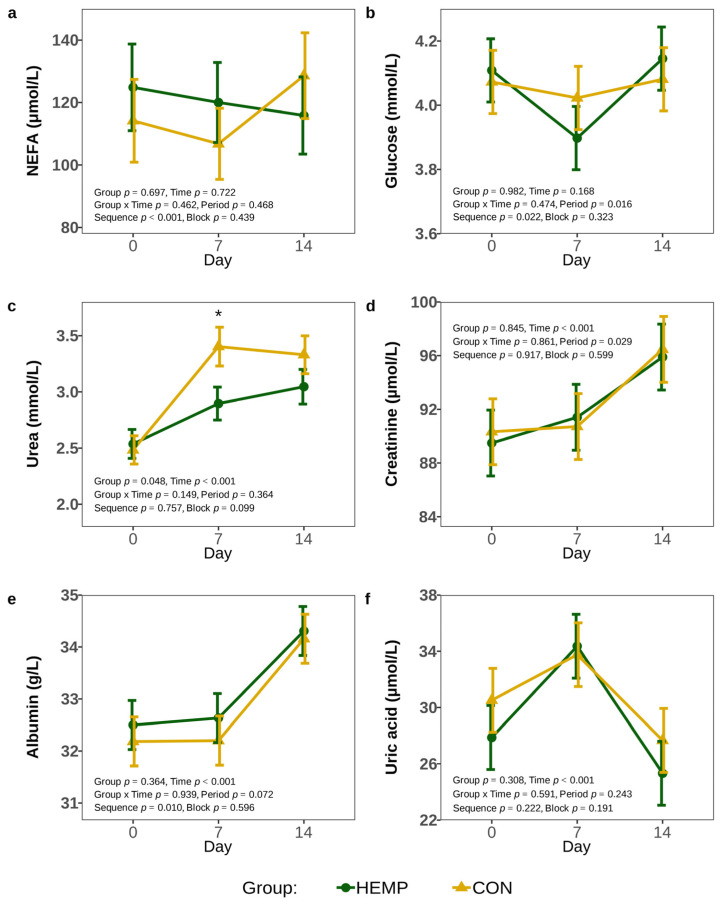
Plasma non-esterified fatty acids (**a**), glucose (**b**), urea (**c**), creatinine (**d**), albumin (**e**), and uric acid (**f**) concentration of cows fed a diet containing 7.4% hemp leaves (HEMP, *n* = 12) or 3.5% soya extraction meal (CON, *n* = 12) on day 0, 7 and 14. For statistical analysis of the NEFA concentration, a log-gamma generalized mixed model was used and the data were back-transformed for interpretation. The urea concentration was transformed using log for statistical analysis and back- transformed for interpretation. * *p* < 0.05.

To investigate if the lower DMI of HEMP cows influenced the post-absorptive metabolism, we analyzed plasma metabolites markers of the energy and protein metabolism. Plasma NEFA, glucose, creatinine, uric acid, and albumin concentrations did not differ between feeding groups throughout the experimental period (*p* > 0.1, Figure 6). We found plasma urea concentrations significantly higher in cows of the HEMP than the CON group (*p* < 0.05), but this effect was mainly caused due to the significant difference on d 7 (*p* = 0.017), whereas urea concentrations did not differ on d 0 and d 14 (*p* > 0.1; Figure 6c). Furthermore, plasma urea, but also creatinine and albumin concentrations continuously increased over time, whereas uric acid concentrations peaked on d 7 (*p* = 0.001).

### 3.4. Rumen Fluid Metabolites

We examined if the reference points suggesting a different protein metabolism in HEMP cows can also be detected on the ruminal site. The ammonia concentration of rumen fluid collected on d 14 did not differ between feeding groups (*p* > 0.1), but the pH tended to be lower in the HEMP than the CON group (*p* = 0.056, Table 2). However, the total SCFA concentration, the molar percentage of individual SCFA, as well as the acetic acid/propionic acid ratio in rumen fluid were not affected by the dietary treatment (*p* > 0.1), except the molar iso-valeric acid percentage, which was significantly lower in the HEMP group (*p* = 0.047).

### 3.5. Urine Analysis

To explore potential reasons for the tendency of higher NUE, we analyzed urine excretion. After HEMP feeding for two weeks, urinary urea, creatine, creatinine, and total N concentrations were lower than in CON cows (*p* < 0.05, Table 3). HEMP cows further tended to have a lower urinary hippuric acid (*p* = 0.077), uric acid (*p* = 0.078), and purine (*p* = 0.098) concentrations in urine. However, the urinary allantoin concentration (*p* = 0.132) and the creatine/creatinin ratio were not affected by the diet (*p* = 0.142). The urinary N content was significantly lower in HEMP cows (*p* = 0.009). Mean urine output measured on days 15–18 was not significantly different between groups; however, total N excretion with urine tended to be lower in the HEMP compared to the CON group (*p* = 0.054, Table 3).

### 3.6. Excreta Collection and Digestibility, Indirect Calorimetry, and Methane Production

During the 4 d quantitative excreta collection period in the respiration chamber, the respiratory and heart rates, DMI, ECM, and milk constituents were comparable between feeding groups (*p* > 0.1, Table 4). However, the decline in DMI observed during the stay in the respiration chamber in week 3, relative to the second week of feeding, tended to be lower in the HEMP group compared to the control group (*p* = 0.080). The mBW tended to be lower (*p* = 0.056) in the HEMP compared to the CON group. Furthermore, the mean mRQ, FOX, COX, metabolic HP, and EB did not differ between the groups. However, cows of the HEMP group tended to emit 3% less CH_4_ yield (*p* = 0.068), whilst the CH_4_ intensity was comparable between dietary groups. Urinary N excretion tended to be lower in the HEMP group (*p* = 0.054), whereas fecal N excretion did not differ relative to the CON group (Table 5). Accordingly, the CP degradability but also DM, energy, and OM digestibility was comparable between the HEMP and CON groups.

## 4. Discussion

### 4.1. Animal Health Assessment and Physical Activity

The hypothesis of the present study was that feeding hemp leaves of the Santhica 27 variety, which is very low in THC and CBD concentrations, would not negatively affect health, DMI, and milk performance, but reduce urinary nitrogen excretion and methane production of dairy cows. Feeding hemp silage containing high cannabinoid concentrations can negatively affect dairy cow health, as reflected by decreased heart and respiratory rates [14]. In our study, respiration and heart rates were within physiological limits and did not differ significantly between the CON and HEMP group. Thus, we conclude that supplementing 7.4% Santhica 27 hemp leaves to a ration of dairy cows had no negative effect on animal health.

Yet, feeding Santhica 27 hemp leaves affected some behavioral traits of the cows. We observed a longer resting time in cows fed the HEMP diet. In contrary, Irawan et al. (2024) observed that feeding cows a diet containing 13% SHB compared to 13% alfalfa shortened the lying time of late lactating Jersey cows during the first two weeks after the start of feeding which, however, converged in week 3 and 4 of feeding [8]. The authors attributed the influence on lying time in the first two weeks of their experiment to an increased feed sorting of cows when the diet contained SHB [8]. Similarly to the results in our study, Kleinhenz et al. (2022) observed an increase in lying time of 0.7 h/d over a period of two weeks when steers switched from a diet containing no hemp to a diet containing 25 g industrial hemp [13]. The longer lying time was accompanied by a 3.7-fold lower plasma cortisol concentration, which led the authors to suggest that the prolonged lying time would be indicative of stress reduction and improvement in well-being [13]. However, if this hypothesis applies to feeding Santhica 27 hemp leaves as well, further assessment of stress markers, such as hair cortisol concentration or heart rate variability [33,34], would be necessary in future to ascertain the stress levels experienced by the animals. In addition, the observed prolonged resting time may have been caused also by drowsiness. Wagner et al. (2022) observed behavioral abnormalities when feeding a cannabinoid-rich whole plant silage to Holstein cows, which could include increased fatigue, yawning and a somnolent appearance [14]. However, these effects did not occur when feeding whole plant silage with a low cannabinoid content [14], and no discernible behavioral changes indicative of increased sleepiness were noted in the course of our study. In addition, feeding a single dose of 35 g industrial hemp flowers in gelatin capsules to 10-month-old male castrated Holstein cattle did not affect the animal behavior [12]. The shorter time HEMP cows spent feeding in week 1 can be attributed to their lower feed intake compared to the CON group. Surprisingly, feeding time of the HEMP group increased in week 2 compared to week 1 and converged with the CON group in week 2. However, feed intake was significantly lower in the HEMP compared to CON group, suggesting a slower eating rate or an increased sorting of the feed by the animals, similar to what Irawan et al. (2024) have discussed [8]. Apart from the feeding behavior, cows of the HEMP group spent less time ruminating in week 1 and 2, which can be attributed to the lower DMI of the HEMP group, because rumination time and the amount of feed intake are positively correlated [35].

### 4.2. DMI and Performance in the First Two Weeks of Feeding

Contrary to our hypothesis, we found that supplementing TMR with 7.4% Santhica 27 hemp leaves had a negative effect on DMI during the first two weeks of the feeding period. The reduced DMI could be related to the flavor of the diet, which is a combination of various sensory impressions, including sight, taste, smell and texture. The chopped and dried hemp leaves in our study had a comparable particle size (~1–10 mm) to the soybean extraction meal, suggesting that the texture was similar between HEMP and CON rations. Hemp leaves as a feed component had never been used before in our herd, so the cows were unfamiliar with this taste. Unfamiliar tastes can have a negative effect on feed intake in ruminants [36,37,38], therefore taste neophobia may have contributed to the reduced DMI in the first two weeks in our study. This assumption is also supported by the fact that the feed intake increased from the first to the second week in the HEMP group and that there was no difference in DMI between groups in the third week, in which the animals became more accustomed to the taste. Feed intake of the CON group was also lower in the first than in the second week. However, the animals were already familiar with the taste of soya, as all animals were given a soya-containing starter as calves, so that feed intake was not influenced as strongly as in the HEMP group. Similarly to our findings, Parker et al. (2022) observed a decrease in feed intake in lambs fed a diet containing 20% SHB during the first four weeks of the trial, but no negative effect was observed during the 4–8 week trial period [39]. On the other hand, feeding 10% SHB to lambs had no effect on feed intake during the first four weeks, but increased feed intake compared to the comparative diets containing no or 20% SHB [39]. However, the inclusion of 13% SHB to the diet also had a negative effect on the DMI of late lactating Jersey cows [8]. The authors of both studies [8,39] assumed the strong odor of hemp as a possible reason for the low palatability. Although not directly measured, we have also detected a typical strong odor of the HEMP diet. Hence, the possibility of an influence of volatile organic compounds (VOC) determining the odor of hemp leaves and reducing animals’ feed intake cannot be excluded. Besides VOC, hemp containing cannabinoids could also have influenced feed intake negatively. Wagner et al. (2022) observed that the inclusion of a cannabinoid-rich industrial hemp silage in the TMR of Holstein cows diminished feed intake [14]. Conversely, the inclusion of a silage with very low cannabinoid concentration did not impair feed intake [14], suggesting a critical cannabinoid concentration is necessary to reduce feed intake. The negative impact of Δ9-THC on feed intake has already been demonstrated in ruminants [40]. However, the Santhica 27 hemp leaves have a very low THC (~0.0%) and CBD (0.023%) content [15], suggesting that other cannabinoids present in this variety might have contributed to the reduction in DMI. Moreover, Irawan et al. (2024) and Parker et al. (2022) speculated that flavonoids may account for the observed decline in feed intake [8,39]. Hemp is rich in flavonoids but also polyphenols [41,42], both of which have been demonstrated to exert a detrimental impact on feed intake [43,44]. However, feeding pellets containing 43% green hemp biomass with a 1.66-fold higher polyphenol content compared to the control group did not reduce feed intake in sheep [45], indicating no role of hemp flavonoids on feed intake regulation. The lower DMI of the HEMP group was accompanied by a reduced intake of drinking water. Because feed intake and water intake are directly correlated [46,47], the reduction in water intake is presumably a consequence of the lower DMI of HEMP cows.

The reduction in feed, and thus energy and nutrient intake, in the HEMP group resulted in a reduction in milk yield in week 1 and 2, as well as in a trend for a lower milk fat and lactose content in week 2. Accordingly, feeding cows the hemp-containing diet decreased ECM yield in week 2. In contrast, feeding 250 g/day of hemp hay to dairy goats increased the milk yield, but did not affect the milk composition [48]. However, the inclusion of 20 g/day of hemp inflorescences to dairy goats did not affect milk yield or milk components [49]. Similarly to our findings, Irawan et al. (2024) also reported a tendency for a lower milk fat concentration in Jersey cows fed a diet containing 13% SHB, but milk yield and ECM were not compromised, despite a reduction in DMI in these animals [8]. The reason why feeding hemp products with low cannabinoid concentrations to dairy cows reduced the milk fat concentration after at least 1 week of feeding was likely due to the lower energy intake. On the other hand, the lower DMI in the HEMP group did not affect plasma NEFA concentrations whose decline could have reduced the supply of long-chain fatty acids for milk triglyceride synthesis. Rather, it appears that, along with the reduction in DMI, the amount of rumen-derived SCFA serving for de novo milk fatty acid synthesis was diminished. Based on the milk fatty acid profile, Irawan et al. (2024) also assumed a decrease in de novo milk fatty acid synthesis with 13% SHB feeding [8]. To confirm this assumption for Santhica 27 feeding, the milk fatty acid profile should be analyzed in future studies.

### 4.3. Energy and N Utilization Efficiencies and Urinary N

The HEMP group of cows produced comparable amounts of ECM as the CON group despite the lower energy and CP intake in week 1. This resulted in a temporary improved EUE in week 1, which no longer existed thereafter. Furthermore, diet tended to affect NUE. Similarly, Irawan et al. (2024) observed a comparable ECM/DMI ratio and a 3.7% greater NUE in Jersey cows supplemented with 13% SHB compared to alfalfa during a feeding period of 28 d [8]. The greater NUE in cows fed hemp products could be facilitated by the lower excretion of urinary N, whereas the fecal N output was not affected by the diet in the present and former studies [8]. Moreover, sheep fed pellets containing 43% green hemp biomass revealed a reduced urinary but not fecal N output while the N intake remained unaltered relative to controls [45]. By contrast, the lower N intake on d 14 in our study could have caused lower urinary urea, uric acid, creatine, creatinine, and purine concentrations; thus, lower total urinary N excretion. Interestingly, feeding 13% SHB to Jersey cows did not affect N intake but reduced urinary N excretion, primarily due to a lower urine volume and not due to a decrease in urinary N metabolite concentration [8]. The authors suggested an antidiuretic effect of hemp feeding [8]; however, in our study, we did not observe an antidiuretic effect as urine volume did not differ between the groups. Therefore, multiple mechanisms must exist commonly, leading to a reduction in urinary N excretion and an improved NUE with hemp-feed products. Finally, the decline in NUE and EUE from weeks 1 to 2 calls for a critical handling of these efficiency parameters and should only be applied after at least two or more weeks of feeding when animals have completely adapted to the diet.

### 4.4. Plasma Metabolite Concentrations

Cows were not fully adapted to both the HEMP and CON diets after only 7 d of feeding, as indicated by the significant effect of time for creatinine, albumin, urea, and uric acid concentrations changing between d 7 and d 14 of feeding. However, plasma urea concentrations were lower in the HEMP than the CON fed group, particularly on d 7, indicating that HEMP cows had reduced ruminal protein degradation, which in turn is presumably due to the lower N intake. Irrespective of the lower DMI, hemp leaf proteins could also be less ruminally degraded compared to soybean protein, but this assumption needs to be validated in further experiments. Plasma parameters related to energy metabolism, e.g., glucose and NEFA, were not affected by diet or time. Comparably, none of the previous studies investigating the effect of feeding hemp to ruminants reported an effect on plasma glucose and NEFA concentration [8,10,39]. Unchanged plasma NEFA concentrations over time indicate no differences in lipomobilisation, which could have occurred in response to insufficient energy intake in the HEMP group, but the lower DMI in HEMP cows was primarily compensated by a reduction in ECM. However, although an increase in plasma NEFA concentration was not detectable, the RFD tended to decline two weeks after HEMP feeding. Thus, we cannot exclude that unchanged plasma NEFA concentration is a result of lipomobilisation and increased long-chain fatty acid oxidation, both occurring simultaneously under conditions of energy deficiency [50]. In contrast to RFT, BCS did not change over time in HEMP cows, which was similarly observed in SHB versus CON cows fed for 28 d [8]. It must be noted that the correlation between BCS and RFT is not very high when RFD values are below 10 mm [51], which was the case in our study and could explain the different statistical outcomes for BCS and RFT in week 2.

### 4.5. Rumen Fermentation

Diet composition exerts a major impact on the rumen environment influencing, among others, microbial protein synthesis, SCFA production, and pH. Feeding the HEMP diet compared to the CON diet to cows resulted in a lower rumen pH, which can be attributed to the numerical higher SCFA concentration [52]. In addition, the reduced rumination time in the HEMP group may have decreased ruminal pH. During rumination, an increased secretion and subsequent swallowing of saliva occurs. Saliva is rich in hydrogen phosphate and bicarbonate which buffer the rumen pH [53]. Thus, the reduced rumination time likely decreased saliva production, decreasing the buffering capacity and finally ruminal pH. Furthermore, the ruminal pH is influenced by the absorption of SCFA. Feed restriction decreases the absorption rate of SCFA [54], thus the reduced DMI in HEMP cows could have caused reduced absorption of SCFA, which further contributed to a lower pH. Despite the minor drop in pH, due to the HEMP feeding, the pH was within the physiological range [55], thus no impairment of microbial fermentation can be assumed. The observed decrease in rumen pH is inconsistent with previous observations. The feeding of industrial hemp ethanol extraction by-products to Holstein cows did not affect ruminal pH, but in contrast to our findings, total VFA and butyrate concentrations were decreased [10]. In the present study, HEMP feeding decreased the molar proportion of iso-valeric acid, while the molar proportions of the major and other minor SCFAs were unaffected. Hemp contains numerous bioactive secondary plant compounds such as phenols, tannins, flavonoids and cannabinoids [56], which are known to affect the rumen microbial metabolism and microbiome [43,57]. The hemp compounds that account for reduction in iso-valeric acid production have not yet been determined.

Ruminal ammonia is produced by the microbial degradation of feed CP and serves a significant N source for microbial protein synthesis or post-absorptive urea synthesis [58,59]. In the present study, the ruminal ammonia concentration was not affected by HEMP, despite the lower feed and thus CP intake, suggesting comparable ruminal degradation of dietary CP as in the CON group. On the other hand, we found urinary purine concentrations in HEMP cows tended to be lower, suggesting less microbial synthesis in these animals [60]. Although we always sampled rumen fluid at the same time after the morning feeding, the amount of feed ingested, which was not measured, may have been not proportional to the daily DMI of the feeding groups. In such cases, the ruminal ammonia concentration of our HEMP cows might be overestimated. In line with this assumption, Stevens et al. (2022) reported that feeding pellets containing 42% green hemp biomass had a negative effect on ruminal ammonia N concentration in sheep [45]. The authors suggested that hemp polyphenols increased the amount of ruminally ungradable protein [45]. However, Wang et al. (2023) reported a trend towards increased ammonia-N concentration by the inclusion of 6% or 11% industrial hemp ethanol extraction by-product in the TMR of Holstein cows [10]. Taken together, the results show that the influence on ruminal ammonia concentration and microbial protein synthesis strongly depends on the type of hemp product and its constituent ingredients.

### 4.6. Apparent Digestibility and Energy Metabolism During Respiration Chamber Housing

When cows were transferred from the free-ranging barn to the respiration chamber facility, DMI decreased, but the decline was lower in the HEMP than in the CON group, resulting in a comparable DMI of groups in week 3. The lower decline in DMI in the HEMP group suggests that hemp leaves feed attenuates the stress response or helps cows cope with stressful situations. The stress-reducing effect could be explained by the presence of cannabinoids in Santhica 27 hemp leaves. For example, CBD exerted anxiolytic effects in stressed mice [61] and attenuates the response to restraint stress in rats [62]. In addition, Kleinhenz et al. (2022) suggested a stress-reducing effect of cannabidiolic acid (CBDA) in cows [13]. However, examining this hypothesis necessitates the assessment of stress markers, such as hair cortisol concentration or heart rate variability [33,34], which could unfortunately not be measured in the present study.

The comparable feed intake in week 3 resulted in a comparable ECM and thus EB. We observed a comparable apparent CP, DM, energy, and OM digestibility between the HEMP and CON diets. Comparably, feeding a diet containing 13% SHB to late-lactation Jersey cows or 42% green hemp biomass feed to merino ewes also had no effect on DM and OM digestibility or CP degradability [8,45].

It has been shown in in vitro studies that hemp flowers, hemp seeds and their co-products may reduce methane production by the action of various compounds, including flavonoids, unsaturated fatty acids, or acidic cannabinoids [11,63,64]. Our in vivo study demonstrates that feeding almost THC-free hemp leaves to ruminants does not affect CH_4_ production and diminishes CH_4_ yield only by 3%. This result is comparable to the findings of an earlier study in which feeding a TMR containing 13% SHB to Jersey cows did not reduce CH_4_ emissions after 24 d of feeding [8].

Differences in effective concentrations of hemp products used in vitro and in vivo studies may account for contrary results.

### 4.7. Limitations

In the present study, we observed significant period, sequence, and block effects for several of the analyzed parameters. These could be due to unknown environmental influences which may have influenced the results in this study. Our study investigates the short-term effects of feeding industrial hemp leaves to dairy cows. Therefore, only limited conclusions can be drawn about the effects of long-term feeding of hemp leaves, as long-term feeding could result in further adaptations in ruminal digestion. To ensure the adaptation of the rumen microflora to the novel diet, further studies with a longer feeding period are required.

In the present study, the animals were kept in the respiration chamber during week 3. It is possible that, despite the animals being carefully habituated, the experimental results were influenced by stress.

A further limitation of the present study is that only animals in the first lactation were used. In future studies, the inclusion of animals with higher lactation numbers is recommended.

## 5. Conclusions

Dried hemp leaves of the variety Santhica 27 are a suitable source of protein for dairy cows, as the inclusion of hemp in the diet has no negative effects on animal health and does not affect apparent DM, energy, and OM digestibility, or CP degradability. In addition, feeding Santhica 27 hemp leaves to cows enhance the animals’ capacity to cope with stressful circumstances. However, implementation of 7% of these hemp leaves in the diet reduces feed intake and milk yield, which can possibly be avoided by slowly familiarizing the animals with the feed. However, substituting soybean extraction meal with hemp reduces urinary N excretion, which may contribute to reducing nitrogen emissions in dairy production.

## Figures and Tables

**Table 1:** Feed constituents, nutrient composition and energy content of diet (mean ± SD).

Item	Hemp-Containing Diet		Soya-Containing Diet		Washout Diet	
Component, g/kg of DM	Mean	SD	Mean	SD	Mean	SD
Grass–silage	201.05	56.67	208.17	57.70	222.51	42.69
Corn silage	395.57	47.22	410.47	51.18	346.54	32.11
Straw			17.71	0.29	11.88	7.30
Rapeseed extraction meal	87.13	6.76	77.66	9.65	67.47	14.72
Soybean extraction meal			35.06	3.11		
Wheat seeds	24.00	7.86	24.84	7.97	21.88	8.90
Corn meal	61.24	6.24	63.54	6.81	54.31	6.00
Vicia faba	30.51	4.28	31.67	4.60	26.99	3.59
Lupin beans	15.89	11.28	16.54	11.75	13.52	9.82
Concentrate mix ^1^	99.04	4.28	102.68	3.81	186.36	21.53
Mineral feed ^2^	6.22	0.57	6.45	0.55	5.59	1.02
Hemp leaves	74.32	0.85				
Limestone ^3^	3.31	0.20	3.43	0.19	2.97	0.47
Soybean oil	1.72	0.19	1.78	0.19	1.55	0.31
Hay					8.43	8.47
Nutrients, g/kg of DM						
Crude ash	72.83	4.41	64.33	4.11	68.01	4.99
Crude protein	154.17	5.37	152.17	4.41	152.32	4.33
Crude fat	36.17	1.34	34.33	3.09	32.87	8.96
Crude fiber	161.33	4.71	173.33	17.33	171.18	7.20
ADF om	188.83	5.98	200.00	10.60	198.96	2.78
aNDF om	352.83	15.86	358.17	25.15	369.17	17.37
Starch	244.00	18.66	245.67	32.71	244.45	24.93
DM content, %	41.68	1.82	42.01	2.25	41.79	2.78
ME, MJ/kg DM	11.45	0.15	11.45	0.30	11.57	0.10
NEL, MJ/kg DM	7.02	0.11	6.97	0.21	7.09	0.11
GE, J/g DM	18,257	428	18,647	161	18,694	231

ADF om = Acid detergent fiber determined on an organic matter basis, aNDF om = Neutral detergent fiber determined on an organic matter basis, ME = Metabolisable energy, NEL = Net energy for lactation, GE = Gross energy. ^1^ MF2000 (Ceravis Produktion und Transport GmbH, Malchin, Germany): composition: 24% crude protein, 2.6% crude fat, 5.1% crude fiber, 8% crude ash, 0.73% calcium, 0.5% phosphorus, 0.65% sodium, 7.1 MJ NEL/kg. Additives: 10,000 I.E. vitamin A, 1125 I.E. vitamin D3, 40 mg vitamin E, 0.6 mg I, 0.4 mg Co, 50 mg Mn, 75 mg Zn, 0.4 mg Se. ^2^ Panto Mineral R 8609 (HL Hamburger Leistungsfutter GmbH, Hamburg, Germany): composition: 20% calcium, 6% phosphorous, 8% sodium, 6% magnesium, 0.03% inorganic nitrogen, 13.7% phosphorous pentoxide. Additives per kg original substance: 900,000 IU vitamin A, 200,000 IU vitamin D3, 4.5 g vitamin E, 1.5 g Cu, 8 g Zn, 5 g Mn, 60 mg I, 21 mg Co, 50 mg Se. ^3^ Bergophor CaCO3 V001 (Hohburg Mineralfutter GmbH, Lossatal, Germany): 37% calcium.

**Table 2:** Ruminal pH, NH_3_-concentration, total SCFA, and molar percentage of individual SCFA of cows fed a diet containing 7.4% hemp leaves (HEMP, *n* = 12) or 3.5% soya extraction meal (CON, *n* = 12) on d 14 of the feeding period.

	HEMP		CON		*p* Values			
	EMM	SE	EMM	SE	Group	Period	Sequence	Block
pH	6.52	0.084	6.74	0.084	0.056	0.233	0.141	0.494
NH_3_, mmol/L	5.77	0.618	6.53	0.618	0.383	0.671	0.268	0.182
Total SCFA, mmol/L	135.0	7.58	127.0	7.58	0.432	0.273	0.454	0.041
Molar percentage of total SCFA, mol%								
Acetic acid	61.00	0.703	62.20	0.703	0.159	0.675	0.068	0.974
Propionic acid	22.80	0.798	21.50	0.798	0.166	0.358	0.010	0.355
iso-butyric acid	0.83	0.029	0.86	0.029	0.405	0.423	0.122	0.057
*n*-butyric acid	12.00	0.270	11.90	0.270	0.757	0.053	0.961	0.032
iso-valeric acid	1.09	0.063	1.25	0.063	0.047	0.026	0.971	0.272
*n*-valeric acid	1.64	0.051	1.53	0.051	0.152	0.318	0.414	0.336
*n*-caproic acid	0.65	0.040	0.73	0.040	0.176	0.111	0.147	0.111
Acetic acid/Propionic acid ratio	2.74	0.12	2.93	0.12	0.166	0.468	0.077	0.644

EMM = estimated marginal mean, SCFA = short-chain fatty acids.

**Table 3:** Urinary N metabolite concentrations of cows fed a diet containing 7.4% hemp leaves (HEMP, *n* = 12, except for urine quantity and urinary N excretion *n* = 9) or 3.5% soya extraction meal (CON, *n* = 12, except for urine quantity and urinary N excretion *n* = 10) on d 14 of the feeding period.

	HEMP		CON			*p* Values		
	EMM	SE	EMM	SE	Group	Period	Sequence	Block
Urea, g/L	10.3	1.04	14.4	1.04	0.018	0.306	0.839	0.100
Uric acid, mg/L	0.43	0.056	0.54	0.056	0.078	0.028	0.420	0.712
Allantoin, mg/L	3.64	0.297	4.30	0.297	0.132	0.389	0.435	<0.001
Purine derivates, mg/L	4.07	0.317	4.85	0.317	0.098	0.338	0.365	<0.001
Creatine, mg/L	0.63	0.069	0.95	0.069	0.004	0.460	0.132	0.546
Creatinine, mg/L	0.76	0.059	0.96	0.059	0.025	0.742	0.947	0.118
Hippuric acid, mg/L	12.9	1.13	15.7	1.13	0.077	0.517	0.985	0.880
Creatine/ creatinine ratio	0.74	0.049	0.84	0.049	0.142	0.815	0.025	0.080
N, wt%	11.8	0.39	13.6	0.39	0.001	0.250	0.863	0.053
Urine quantity, kg	11.5	0.46	11.1	0.45	0.401	0.172	0.352	0.151
Urinary N excretion, g/d	93.2	8.69	116.1	8.37	0.054	0.045	0.696	0.203

EMM = estimated marginal mean.

**Table 4:** Respiratory rate, heart rate, DMI, mBW, ECM, milk, and energy variables of cows fed a diet containing 7.4% hemp leaves (HEMP, *n* = 10, except for the milk constitutes *n* = 9) or 3.5% soya extraction meal (CON, *n* = 11) determined during the stay in the respiration chamber (d 15 to 18 of the feeding period).

	HEMP		CON		*p* Values			
Item	EMM	SE	EMM	SE	Group	Sequence	Period	Block
Respiratory rate (min^−1^)	34	1.4	34	1.3	0.625	0.375	0.140	0.016
Heart rate (min^−1^)	75	1.7	75	1.6	0.834	0.508	0.978	0.017
mBW, kg^0.75^	124	1.88	127	1.85	0.056	0.002	0.975	0.167
DMI, kg/d	17.6	0.53	17.4	0.51	0.658	0.826	0.182	0.113
DMI decline, % ^1^	12.7	3.3	18.5	3.1	0.080	0.329	0.508	0.084
Milk yield, kg/d	29.8	1.39	29.7	1.37	0.846	0.581	0.911	0.729
ECM, kg/d	29.5	1.16	30.1	1.09	0.526	0.577	0.874	0.510
Milk protein	3.63	0.058	3.59	0.057	0.130	0.385	0.001	0.609
Milk fat, %	4.06	0.256	4.23	0.251	0.195	0.781	0.384	0.949
Lactose, %	4.99	0.038	4.99	0.035	0.992	0.399	0.295	0.096
Milk urea, mg/L	232	13.0	238	11.6	0.747	0.255	0.0.34	0.264
mRQ	0.92	0.008	0.91	0.007	0.243	0.402	0.010	0.119
FOX/mBW, g/kg^0.75^	5.7	0.71	6.9	0.66	0.268	0.323	0.003	0.212
COX/mBW, g/kg^0.75^	48.7	2.26	46.6	2.15	0.371	0.665	0.063	0.241
HP/mBW, kJ/kg^0.75^	1135	21.1	1138	22.8	0.849	0.754	0.026	0.314
EB, MJ ME	−32.7	5.73	−38.1	5.11	0.534	0.727	0.351	0.036
CH_4,_ L/d	511	14.4	522	14.1	0.284	0.584	0.042	0.386
CH_4_/DMI, L/kg	29.2	0.80	30.2	0.79	0.068	0.814	0.538	0.422
CH_4_/ECM, L/kg	17.4	0.68	17.5	0.63	0.865	0.907	0.237	0.850

EMM = estimated marginal mean, mBW = metabolic body weight, DMI dry matter intake, ECM = energy corrected milk yield, mRQ = metabolic respiratory quotient, FOX = fat oxidation, COX = carbohydrate oxidation, HP = heat production, EB = energy balance, ^1^ DMI decline, decline in DMI during the 4 d measurement period relative to the preceding feeding week in the free-stall barn.

**Table 5:** Urinary and fecal N excretions and the apparent DM, energy and OM digestibility, and CP degradability of cows fed a diet containing 7.4% hemp leaves (HEMP, *n* = 10, except for urinary N excretion *n* = 9) or 3.5% soya extraction meal (CON, *n* = 11, except for urinary N excretion *n* = 10) determined during the stay in the respiration chamber (d 15 to 18 of the feeding period).

	HEMP		CON		*p* Values			
	EMM	SE	EMM	SE	Group	Period	Sequence	Block
Urinary N excretion, g/d	93.2	8.69	116.1	8.37	0.054	0.045	0.696	0.203
Fecal N excretion, g/d	151.0	6.74	147.0	6.32	0.549	0.715	0.348	0.036
Crude protein degradability, %	64.1	1.33	65.2	1.25	0.590	0.247	0.368	0.140
OM digestibility, %	67.4	1.46	58,9	1.37	0.507	0.324	0.722	0.039
DM digestibility, %	65.7	1.56	67.5	1.46	0.407	0.353	0.875	0.036
Energy digestibility, %	65.1	1.73	67.1	1.62	0.384	0.228	0.800	0.061

EMM = estimated marginal mean, OM = organic matter, DM = dry matter.

## Data Availability

As further project results are the subject of other publications that are currently under review, the data are available on request from the co-author but are not yet stored in a repository.

## Supplementary Materials

The following supporting information can be downloaded at: https://www.mdpi.com/article/10.3390/ani15111662/s1, Figure S1. Twelve dairy cows in the first lactation were fed two isoenergetic and isonitrogenous diets for ad libitum intake for 3 weeks in a crossover design. Cows were randomly assigned to a group prior the trial. In feeding period 1, cows of the HEMP group received a TMR supplemented with 7.4 % (on the DM basis) THC-free dried hemp leaves. In parallel, the CON group received a TMR containing 3.5 % soya extract meal. Both groups were fed a hemp- and soy-free TMR during the subsequent 2-week washout period. In the following feeding period 2, the cows were fed the reverse diet for a further 3 weeks. In each feeding period, cows were kept in the free-ranging barn for 2 weeks and housed in a respiration chamber for a further 4 day-measurement period. Blood samples were taken on day 0, 7, and 14. A urine sample and a rumen fluid sample were collected on day 14 of each feeding period.

## Abbreviations

The following abbreviations are used in this manuscript:

BFT	back fat thickness
BW	body weight
CBD	cannabidiol
CO2 ferm	CO2 from fermentative and metabolic () processes
CON	control-group, receiving a TMR containing 3.5% soya extract meal
COX	carbohydrate oxidation
CP	crude protein
d	day
DM	dry matter
DMI	dry matter intake
EB	Energy balance
ECM	energy corrected milk yield
EUE	feed energy utilization efficiency
FOX	fat oxidation
HEMP	group receiving a TMR containing 7.4% dried industrial hemp leaves of the low THC variety “Santhica 27”
HP	heat production
HPLC	high-performance liquid chromatography
mBW	metabolic body weight
mCO2	CO2 from metabolic processes
MEI	metabolizable energy intake
mRQ	metabolic respiratory quotient
N	nitrogen
NEFA	non-esterified fatty acids
Nu	urinary N excretion
NUE	nitrogen utilization efficiency
OM	organic matter
SCFA	short-chain fatty acids
SD	standard deviation
SE	standard errors
SHB	spent hemp biomass
THC	delta-9 tetrahydrocannabinol.
TMR	total mixed ration

## References

[1] Janson M. Woher Kommen Unsere Sojabohnen?. https://de.statista.com/infografik/31782/deutsche-importe-von-sojabohnen-nach-herkunftslaendern/.

[2] Escobar N., Tizado E.J., zu Ermgassen E.K.H.J., Löfgren P., Börner J., Godar J. (2020). Spatially-explicit footprints of agricultural commodities: Mapping carbon emissions embodied in Brazil’s soy exports. Glob. Environ. Chang..

[3] Semwogerere F., Katiyatiya C.L.F., Chikwanha O.C., Marufu M.C., Mapiye C. (2020). Bioavailability and Bioefficacy of Hemp By-Products in Ruminant Meat Production and Preservation: A Review. Front. Vet. Sci..

[4] Nchama C.N.N., Fabro C., Baldini M., Saccà E., Foletto V., Piasentier E., Sepulcri A., Corazzin M. (2022). Hempseed By-Product in Diets of Italian Simmental Cull Dairy Cows and Its Effects on Animal Performance and Meat Quality. Animals.

[5] Wang Y., Gao J., Cheng C., Lv J., Lambo M.T., Zhang G., Li Y., Zhang Y. (2022). Nutritional Values of Industrial Hemp Byproducts for Dairy Cattle. Animals.

[6] Baldini M., Da Borso F., Rossi A., Taverna M., Bovolenta S., Piasentier E., Corazzin M. (2020). Environmental Sustainability Assessment of Dairy Farms Rearing the Italian Simmental Dual-Purpose Breed. Animals.

[7] Helstad A., Forsén E., Ahlström C., Labba I.C.M., Sandberg A., Rayner M., Purhagen J.K. (2022). Protein extraction from cold-pressed hempseed press cake: From laboratory to pilot scale. J. Food Sci..

[8] Irawan A., Puerto-Hernandez G.M., Ford H.R., Busato S., Ates S., Cruickshank J., Ranches J., Estill C.T., Trevisi E., Bionaz M. (2024). Feeding spent hemp biomass to lactating dairy cows: Effects on performance, milk components and quality, blood parameters, and nitrogen metabolism. J. Dairy Sci..

[9] Kleinhenz M.D., Magnin G., Ensley S.M., Griffin J.J., Goeser J., Lynch E., Coetzee J.F. (2020). Nutrient concentrations, digestibility, and cannabinoid concentrations of industrial hemp plant components. Appl. Anim. Sci..

[10] Wang Y., Yu Q., Wang X., Song J., Lambo M.T., Huang J., He P., Li Y., Zhang Y. (2023). Replacing alfalfa hay with industrial hemp ethanol extraction byproduct and Chinese wildrye hay: Effects on lactation performance, plasma metabolites, and bacterial communities in Holstein cows. Front. Vet. Sci..

[11] Jensen R.H., Rønn M., Thorsteinsson M., Olijhoek D.W., Nielsen M.O., Nørskov N.P. (2022). Untargeted Metabolomics Combined with Solid Phase Fractionation for Systematic Characterization of Bioactive Compounds in Hemp with Methane Mitigation Potential. Metabolites.

[12] Kleinhenz M.D., Magnin G., Lin Z., Griffin J., Kleinhenz K.E., Montgomery S., Curtis A., Martin M., Coetzee J.F. (2020). Plasma concentrations of eleven cannabinoids in cattle following oral administration of industrial hemp (*Cannabis sativa*). Sci. Rep..

[13] Kleinhenz M.D., Weeder M., Montgomery S., Martin M., Curtis A., Magnin G., Lin Z., Griffin J., Coetzee J.F. (2022). Short term feeding of industrial hemp with a high cannabidiolic acid (CBDA) content increases lying behavior and reduces biomarkers of stress and inflammation in Holstein steers. Sci. Rep..

[14] Wagner B., Gerletti P., Fürst P., Keuth O., Bernsmann T., Martin A., Schäfer B., Numata J., Lorenzen M.C., Pieper R. (2022). Transfer of cannabinoids into the milk of dairy cows fed with industrial hemp could lead to Δ9-THC exposure that exceeds acute reference dose. Nat. Food.

[15] Tsaliki E., Kalivas A., Jankauskiene Z., Irakli M., Cook C., Grigoriadis I., Panoras I., Vasilakoglou I., Dhima K. (2021). Fibre and Seed Productivity of Industrial Hemp (*Cannabis sativa* L.) Varieties under Mediterranean Conditions. Agronomy.

[16] Edmonson A.J., Lean I.J., Weaver L.D., Farver T., Webster G. (1989). A Body Condition Scoring Chart for Holstein Dairy Cows. J. Dairy Sci..

[17] Schröder U.J., Staufenbiel R. (2006). Invited Review: Methods to Determine Body Fat Reserves in the Dairy Cow with Special Regard to Ultrasonographic Measurement of Backfat Thickness. J. Dairy Sci..

[18] Muizelaar W., Bani P., Kuhla B., Larsen M., Tapio I., Yáñez-Ruiz D., van Gastelen S., Viereck G., Kuhla B., Danesh Mesgaran S. (2020). Rumen fluid sampling via oral stomach tubing method. Methods in Cattle Physiology and Behaviour Research—Recommendations from the SmartCow Consortium.

[19] Derno M., Elsner H.-G., Paetow E.-A., Scholze H., Schweigel M. (2009). Technical note: A new facility for continuous respiration measurements in lactating cows. J. Dairy Sci..

[20] Müller C.B.M., Görs S., Derno M., Tuchscherer A., Wimmers K., Zeyner A., Kuhla B. (2021). Differences between Holstein dairy cows in renal clearance rate of urea affect milk urea concentration and the relationship between milk urea and urinary nitrogen excretion. Sci. Total Environ..

[21] Prahl M.C., Müller C.B.M., Wimmers K., Kuhla B. (2023). Mammary gland, kidney and rumen urea and uric acid transporters of dairy cows differing in milk urea concentration. Sci. Rep..

[22] Erdmann S., Derno M., Schäff C.T., Börner S., Kautzsch U., Kuhla B., Hammon H.M., Tuchscherer A., Röntgen M. (2019). Comparative analyses of estimated and calorimetrically determined energy balance in high-yielding dairy cows. J. Dairy Sci..

[23] Chwalibog A., Jensen K., Thorbek G. (1996). Oxidation of nutrients in bull calves treated with beta-adrenergic agonists. Arch. Tierernahr..

[24] Frayn K.N. (1983). Calculation of substrate oxidation rates in vivo from gaseous exchange. J. Appl. Physiol..

[25] Brouwer E., Blaxter K.L. (1965). Report of sub-committee on constants and factors. Proceedings of the 3rd EAAP Symposium on Energy Metabolism, Troon, Scotland, May 1964.

[26] Labes D., Schütz H., Lang B. (2022). *PowerTOST: Power and Sample Size for (Bio)Equivalence Studies*, Version 1.5-4. https://cran.r-project.org/web/packages/PowerTOST/index.html.

[27] Champely S., Ekstrom C., Dalgaard P., Gill J., Weibelzahl S., Anandkumar A., Ford C., Volcic R., De Rosario H. (2020). *PWR: Basic Functions for Power Analysis*, R package Version 1.3-0. https://CRAN.R-project.org/package=pwr.

[28] R Core Team (2023). R: A Language and Environment for Statistical Computing.

[29] Lüdecke D., Ben-Shachar M.S., Patil I., Waggoner P., Makowski D. (2021). Performance: An R Package for Assessment, Comparison and Testing of Statistical Models. J. Open Source Softw..

[30] Kuznetsova A., Brockhoff P.B., Christensen R.H.B. (2017). lmerTest Package: Tests in Linear Mixed Effects Models. J. Stat. Softw..

[31] Brooks M.E., Kristensen K., van Benthem K.J., Magnusson A., Berg C.W., Nielsen A., Skaug H.J., Mächler M., Bolker B.M. (2017). glmmTMB Balances Speed and Flexibility Among Packages for Zero-inflated Generalized Linear Mixed Modeling. R J..

[32] Schielzeth H., Dingemanse N.J., Nakagawa S., Westneat D.F., Allegue H., Teplitsky C., Réale D., Dochtermann N.A., Garamszegi L.Z., Araya-Ajoy Y.G. (2020). Robustness of linear mixed-effects models to violations of distributional assumptions. Methods Ecol. Evol..

[33] Grelet C., Dries V.V., Leblois J., Wavreille J., Mirabito L., Soyeurt H., Franceschini S., Gengler N., Brostaux Y., Consortium H. (2022). Identification of chronic stress biomarkers in dairy cows. Animal.

[34] Mohr E., Langbein J., Nürnberg G. (2002). Heart rate variability: A noninvasive approach to measure stress in calves and cows. Physiol. Behav..

[35] Schirmann K., Chapinal N., Weary D.M., Heuwieser W., von Keyserlingk M.A.G. (2012). Rumination and its relationship to feeding and lying behavior in Holstein dairy cows. J. Dairy Sci..

[36] Costa J.H.C., Daros R.R., von Keyserlingk M.A.G., Weary D.M. (2014). Complex social housing reduces food neophobia in dairy calves. J. Dairy Sci..

[37] Chapple R., Lynch J.J. (1986). Behavioural factors modifying acceptance of supplementary foods by sheep. Res. Dev. Agric..

[38] Launchbaugh K.L., Provenza F.D., Werkmeister M.J. (1997). Overcoming food neophobia in domestic ruminants through addition of a familiar flavor and repeated exposure to novel foods. Appl. Anim. Behav. Sci..

[39] Parker N.B., Bionaz M., Ford H.R., Irawan A., Trevisi E., Ates S. (2022). Assessment of spent hemp biomass as a potential ingredient in ruminant diet: Nutritional quality and effect on performance, meat and carcass quality, and hematological parameters in finishing lambs. J. Anim. Sci..

[40] McLaughlin C.L., Baile C.A., Bender P.E. (1979). Cannabinols and feeding in sheep. Psychopharmacology.

[41] Aloo S.O., Kwame F.O., Oh D.-H. (2023). Identification of possible bioactive compounds and a comparative study on in vitro biological properties of whole hemp seed and stem. Food Biosci..

[42] Axentii M., Codină G.G. (2024). Exploring the Nutritional Potential and Functionality of Hemp and Rapeseed Proteins: A Review on Unveiling Anti-Nutritional Factors, Bioactive Compounds, and Functional Attributes. Plants.

[43] Besharati M., Maggiolino A., Palangi V., Kaya A., Jabbar M., Eseceli H., De Palo P., Lorenzo J.M. (2022). Tannin in Ruminant Nutrition: Review. Molecules.

[44] Zhan J., Liu M., Su X., Zhan K., Zhang C., Zhao G. (2017). Effects of alfalfa flavonoids on the production performance, immune system, and ruminal fermentation of dairy cows. Asian-Australas. J. Anim. Sci..

[45] Stevens S.A., Krebs G.L., Scrivener C.J., Noble G.K., Blake B.L., Dods K.C., May C.D., Tai Z.X., Clayton E.H., Lynch E.E. (2022). Nutrient digestibility, rumen parameters, and (cannabinoid) residues in sheep fed a pelleted diet containing green hemp (*Cannabis sativa* L.) biomass. Transl. Anim. Sci..

[46] Holter J.B., Urban W.E. (1992). Water Partitioning and Intake Prediction in Dry and Lactating Holstein Cows. J. Dairy Sci..

[47] Lukas J.M., Reneau J.K., Linn J.G. (2008). Water Intake and Dry Matter Intake Changes as a Feeding Management Tool and Indicator of Health and Estrus Status in Dairy Cows. J. Dairy Sci..

[48] Iommelli P., Zicarelli F., Amato R., Musco N., Sarubbi F., Bailoni L., Lombardi P., Bennardo F.D., Infascelli F., Tudisco R. (2024). The Effects of Hemp Hay (*Canapa sativa* L.) in the Diets of Grazing Goats on Milk Production and Fatty Acid Profile. Anim..

[49] Amato R., Oteri M., Chiofalo B., Zicarelli F., Musco N., Sarubbi F., Pacifico S., Formato M., Lombardi P., Bennardo F.D. (2024). Diet supplementation with hemp (*Cannabis sativa* L.) inflorescences: Effects on quanti-qualitative milk yield and fatty acid profile on grazing dairy goats. Vet. Q..

[50] Börner S., Albrecht E., Schäff C., Hacke S., Kautzsch U., Derno M., Hammon H.M., Röntgen M., Sauerwein H., Kuhla B. (2013). Reduced AgRP activation in the hypothalamus of cows with high extent of fat mobilization after parturition. Gen. Comp. Endocrinol..

[51] Hussein H.A., Westphal A., Staufenbiel R. (2013). Relationship between body condition score and ultrasound measurement of backfat thickness in multiparous Holstein dairy cows at different production phases. Aust. Vet. J..

[52] Kitkas G.C., Valergakis G.E., Kritsepi-Konstantinou M., Gelasakis A.I., Katsoulos P.D., Kalaitzakis E., Panousis N.K. (2022). Association between Ruminal pH and Rumen Fatty Acids Concentrations of Holstein Cows during the First Half of Lactation. Ruminants.

[53] Beauchemin K.A. (2018). Invited review: Current perspectives on eating and rumination activity in dairy cows. J. Dairy Sci..

[54] Zhang S., Albornoz R.I., Aschenbach J.R., Barreda D.R., Penner G.B. (2013). Short-term feed restriction impairs the absorptive function of the reticulo-rumen and total tract barrier function in beef cattle. J. Anim. Sci..

[55] Dijkstra J., Ellis J.L., Kebreab E., Strathe A.B., López S., France J., Bannink A. (2012). Ruminal pH regulation and nutritional consequences of low pH. Anim. Feed. Sci. Technol..

[56] Radwan M.M., Chandra S., Gul S., ElSohly M.A. (2021). Cannabinoids, Phenolics, Terpenes and Alkaloids of Cannabis. Molecules.

[57] Vasta V., Daghio M., Cappucci A., Buccioni A., Serra A., Viti C., Mele M. (2019). Invited review: Plant polyphenols and rumen microbiota responsible for fatty acid biohydrogenation, fiber digestion, and methane emission: Experimental evidence and methodological approaches. J. Dairy Sci..

[58] Tan Z., Murphy M.R. (2004). Ammonia production, ammonia absorption, and urea recycling in ruminants. A review. J. Anim. Feed. Sci..

[59] Breves G., Diener M., Gäbel G., Breves G., Diener M., Gäbel G. (2022). Vormägen. Physiologie der Haustiere.

[60] Tas B.M., Susenbeth A. (2007). Urinary purine derivates excretion as an indicator of in vivo microbial N flow in cattle: A review. Livest. Sci..

[61] Fogaça M.V., Campos A.C., Coelho L.D., Duman R.S., Guimarães F.S. (2018). The anxiolytic effects of cannabidiol in chronically stressed mice are mediated by the endocannabinoid system: Role of neurogenesis and dendritic remodeling. Neuropharmacology.

[62] Resstel L.B., Tavares R.F., Lisboa S.F., Joca S.R., Corrêa F.M., Guimarães F.S. (2009). 5-HT1A receptors are involved in the cannabidiol-induced attenuation of behavioural and cardiovascular responses to acute restraint stress in rats. Br. J. Pharmacol..

[63] Wang S., Kreuzer M., Braun U., Schwarm A. (2017). Effect of unconventional oilseeds (safflower, poppy, hemp, camelina) on in vitro ruminal methane production and fermentation. J. Sci. Food Agric..

[64] Vastolo A., Calabrò S., Pacifico S., Koura B.I., Cutrignelli M.I. (2021). Chemical and nutritional characteristics of *Cannabis sativa* L. co-products. J. Anim. Physiol. Anim. Nutr..

