# Supplementary material for: Replacing Soybean Meal with Hemp Leaves with Very Low THC Content in the Diet for Dairy Cows: Impact on Digestibility, Nitrogen Use Efficiency and Energy Metabolism
Source: Animals (Basel). 2025 Jun 4;15(11):1662. doi: 10.3390/ani15111662 (PMC12153878; doi:10.3390/ani15111662)
Supplement: Supplementary file 1 [file animals-15-01662-s001.zip › animals-3592716-supplementary.pdf]

Replacing soybean meal with hemp leaves with very low-THC content in the diet for dairy cows: impact on digestibility, nitrogen use efficiency and energy metabolism

Jessica Schwerdtfeger, Solvig Görs, Björn Kuhla

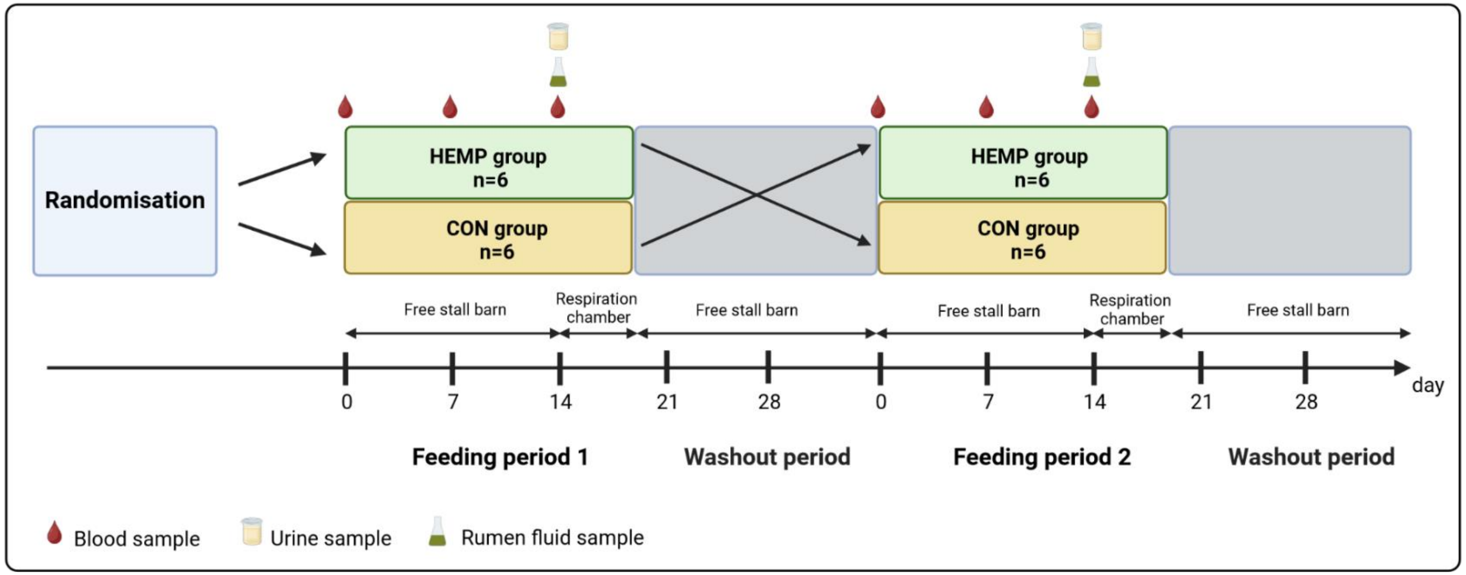

Figure S1. Twelve dairy cows in the first lactation were fed two isoenergetic and isonitrogenous diets for ad libitum intake for 3 weeks in a cross-over design. Cows were randomly assigned to a group prior the trial. In feeding period 1, cows of the HEMP group received a TMR supplemented with 7.4 % (on the DM basis) THC-free dried hemp leaves. In parallel, CON group received a TMR containing 3.5 % soya extract meal. Both groups were fed a hemp- and soy-free TMR during the subsequent 2-week washout period. In the following feeding period 2, cows were fed the reverse diet for further 3 weeks. In each feeding period, cows were kept in the free-ranging barn for 2 weeks and housed in a respiration chamber for a further 4 day-measurement period. Blood samples were taken on day 0, 7 and 14. A urine sample and a rumen fluid sample were collected on day 14 of each feeding period.
